# Anti-quorum sensing activity of *Boerhavia diffusa* against *Pseudomonas aeruginosa* PAO1

**DOI:** 10.6026/97320630019310

**Published:** 2023-03-31

**Authors:** Shravani V, AS Smiline Girija, Himabindu, Madhan Krishnan, Shyamaladevi Babu

**Affiliations:** 1 Department of Microbiology, School of Allied Health Sciences, Mallareddy University, Hyderabad, Telangana, India; 2 Department of Microbiology, Saveetha Dental College and Hospitals, Saveetha Institute of Medical and Technical Sciences (SIMATS), Chennai, India; 3 Department of Microbiology, Mallareddy Institute of Medical Sciences, Hyderabad, Telangana, India; 4 Faculty of Allied Health Sciences, Chettinad Hospital and Research Institute, Chettinad Academy of Research and Education, Kelambakkam-603103, Tamilnadu, India

**Keywords:** *Pseudomonas aeruginosa*, virulence factors, *Boerhavia diffusa*, molecular docking

## Abstract

Quorum sensing (QS) is one of the key virulence factors in *Pseudomonas aeruginosa* and causes recalcitrant infections. Multi-drug resistance and biofilm formation seem to be regulated by cell-to-cell communication system through QS. Thus this study is aimed to assess the efficacy of ethanolic leaf extract of *Boerhavia diffusa* in acting against the QS-regulated virulence traits. Fresh leaves of *B. diffusa* were dried and the ethanolic crude extract was checked for antimicrobial and anti biofilm effect against *P. aeruginosa*. The active components and the biological structures were elucidated by GC-MS, HPLC and NMR analysis respectively. Further, computational analyses were also performed to assess the drug ligand interactions based on the docking scores and binding energy. The results suggested that the MIC concentration showed a significant effect in inhibiting the QS network circuit of *P. aeruginosa*. The docking results showed that leaf had bioactive compounds that exhibit strong binding affinity towards transcriptional activators of the QS circuit in *P. aeruginosa*, i.e., LasR, as compared to the natural ligands, 3-oxo-C12-HSL and C4-HSL. These results clearly depictthe efficacy of *Boerhavia diffusa* and its phytoconstituents as promising QS antagonist which can be further applied in the treatment strategies for the diseases caused by *P. aeruginosa*.

## Background:

*Pseudomonas aeruginosa* belong to the Gram-negative family of bacteria and it is known to be mono-flagellated and possess the characteristic of obligatory aerobic bacteria [1]. It is well known as an escape pathogen [2] and habitats in different diverse kind of environment including soil, plants, hospitals, etc. It is also reported as an opportunistic human pathogen as it sometimes infects healthy persons and also affects the persons with AIDS, cystic fibrosis, cancer and burn victims. *P. aeruginosa* is the root causes of high mortality rate especially in cystic fibrosis condition. This is mainly due to the potent virulence factors such as proteases, hemolysins, exotoxin A, production of pigment like pyocyanin and biofilm formation [1,2, 3,4]. Formation of biofilm is an important mechanism by which inter-cellular communication through the process of quorum sensing (QS) mediated by the auto-inducers (AI). Auto-inducers are described as hormone like molecules that was formed as clusters in the extracellular matrix. At a particular point of saturation, the AIs get captured by their corresponding cognate receptor, activating the regulators towards the modulation and various QS gene expression. This will lead to a consequence of process such as adaptation, colonization, antibiotic resistance, plasmid conjugation, etc. In the family of *P. aeruginosa* the AI category was expressed by LuxI/LuxR - typed proteins. In this system, it is observed that hcnABC operon plays a key role for the production of HCN utilizing HCN synthase. Generally, *P. aeruginosa* have four major Quorum sensing process [5] that works as a group as functional regulators [3] dominating the transcriptional regulation of hcnABC by altering the genes such as LasR, ANR and RhlR [7].

During the host pathogen interactions, different kinds of compound are released which is basically the bacterial auto-inducers, stress hormones & cytokines. The understanding about these QS based interactions were not demonstrated well enough till date. Few reports analyzed about LuxR described that they exist as homo-dimers with presence of two domains. Both these domains are connected by a small linker region [2,6]. Targeting these QS based virulence factors by plant based bio-compounds hold promising to tackle and mitigate *P. aeruginosa*. In this note, *Boerhavia diffusa* that belong to the flowering plant species family is been selected to assess QS inhibiting property in the present study. *B. diffusa* is commonly addressed as Punarnava and various bio-compounds with different kinds of biological activities have been documented [18,19,20, 21]. All their herbs and roots are rich in sources of proteins and fats, its roots rich in and the phytochemical analysis of this plant describes the presence of alkaloids contents as well as a range of amino acids [22]. Thus the present study is designed to assess the QS inhibiting property of *B. diffusa* extract against the LasR protein of *P. aeruginosa* by *in-vitro* and in-silico analysis.

## Materials and methods:

## Bacterial strains and anti biogram profiling:

The clinical strains isolated from different specimens submitted to department of microbiology, Mallareddy Hospitals were used in this study. The test bacterial culture of *P. aeruginosa* PAO1 (MTCC 3541) was procured from Microbial Type Culture Collection and Gene Bank (MTCC), IMTECH, Chandigarh, India. The strains were cultured in Luria Bertani (LB) broth at 37°C, until an optical density (O.D.) of 0.4 was attained at 600 nm. Susceptibility testing against routine antibiotics for the *Pseudomonas aeruginosa* isolates was performed by Kirby Bauer disc diffusion method according to CLSI guidelines (2021) with antibiotics, Ceftazidime (30 µg), Gentamicin (10 µg), Tobramycin (mcg), Piperacillin Tozobactam (mcg), Amikacin (30 mcg), Aztreonam (30 mcg), Cefepime (30 µg), Ciprofloxacin (5 µg), Imipenem (10 µg), Meropenem (10 µg), Doripenem (10 µg) and Levofloxacin (5 µg).

## Plant Material Collection and extract preparation:

Fresh leaves of *Boerhavia diffusa*, *Terminalia chebula*, *Asparagus racemosus*, *Syzgium cumini* and *Azadiracta indica*were obtained from locally available medicinal plant store, Hyderabad. The samples were rinsed with sterile water and kept for drying under shade condition and were ground into coarse powder. The plant extracts were prepared by gently adding 50 g of the fine plant powder to 250 ml of ethanol (1:5 w/v). The mixtures were further incubated with shaking at 28°C for about 48 hrs and after incubation, filtered using Whatmann filter paper No.1, and were completely dried with a rotary vacuum evaporator. The final extracts were re-suspended in dimethyl sulfoxide and were stored at -20°C.

## Elucidation of antimicrobial activity:

The antimicrobial activity assay was done by adapting the agar well diffusion method. The cultures of biofilm producing P. aeruginosabacteria were tested with the plant extract on the lawn culture of the organism on sterile Muller Hinton agar. 100 µl of plant extracts were added to the appropriate wells and the plates were incubated at 37°C for 24 hr. After incubation, the plates were observed for zone of inhibition to monitor the efficacy of the plant extract in treating the microorganism. The control wells were added with normal saline and ethanol.

## Determination of MIC:

All the five plant extracts (1mg/ml) were added with 50 ml LB broth added with 1% *P. aeruginosa* culture individually and were kept 37°C with constant shaking at the speed of 250 rpm. Eventually, 1000 µl of culture broth was taken at a time period of 2 hrs and the densities of the cells were monitored at 600 nm. The MIC values were obtained by the growth curve analysis.

## Characterization of bioactive compounds:

The *Boerhavia diffusa* plant extract was added in 500 ml of sterile distilled water. Further the mixture was consecutively extracted with various solvents such as ethanol. The samples which showed efficient activity were further analysed to identify the bioactive compounds using HPLC, GC-MS and NMR.

## HPLC:

HPLC Size Exclusion SEC column was prepared with poly (2-hydroxyethyl aspartamide) covalently coated silica. The flow ratio is adjusted to 10:1 and is connected to UV-Vis photodiode. 20 µL of the sample to be eluted is selected as sample size. The sample is passes via the inject valve. The system is configured to generate the start signal from the mass spectrometer to record retention times. For size exclusion the isocratic solvent A, which is formic acid is used at a flow rate of 3ml/minute for a period of 20 minutes. The UV-Vis spectrum of 200-640 nm was used to record without any interruption. The standard solutions were prepared and were eluted at 20 µl volume. The peak areas of the 260nm chromatograms are used to generate the calibration curve. The fractions are collected for each elution and stored at -80°C.

## NMR:

Add 100ml of methanol to 2 grams of crushed plant material. The soaked material is centrifuged for 10 minutes at 10000 rpm and supernatant is collected and evaporated. 0.2 ml of TSP solution + 0.3ml of phosphate buffer + 0.2ml of NaN3 are added to 10mg of the plant extract. The mixture is sonicated and centrifuged at 10000 rpm for 10 minutes. 0.6ml of the supernatant is taken for NMR analysis. NMR measurements are done at 298K with 500MHZ NMR. 1H and 13C frequencies of nuclear resonance are set at 500 and 125 MHz. The suppression power of water peak was maintained at 41db at a pulse delay of 2 seconds, 12ppm spectrum width, 9.8 µs pulse time, 128 scan number, 2.72 sec sampling time, 2 sec relaxation time, FID resolution of 0.18Hz. The adjustment of baseline and phase was done manually. The chemical shifts are calibrated using TSP and the spectra are imported for processing of data. The spectrum was integrated to the range of 0.5 to 10 ppm at an interval of 0.2 ppm. The excel data is generated by normalizing the spectrum integral data and then taken into MATLAB.

## GCMS:

GC-MS (QP-ultra 2010, Shimadzu, Japan) analysis was carried out for TMS derivatives using Zebron 5HT capillary column (30x0.32mmx0.25µm) with electron impact (EI) ionization. Helium was used as a carrier gas at 1.57 mL min-1. In GC, injection temperature was maintained at 250ºC. The oven temperature profile was 50°C (2 min hold), increased to 210°C at the rate of 4°C/min and final hold for 18 min in split mode with 2:1 split ratio. In MS, ion source temperature was 220°C and interface temperature was 250°C in scan mode with m/z detection from 45-900.

## Molecular docking analysis:

The *Boerhavia diffusa* phyto compounds with other natural auto inducers such as 3-oxo-C12-HSL and C4-HSL were further performed with molecular docking analysis against the QS receptor protein of *P. aeruginosa*, LasR ligand-binding domain (PDB ID: 2UV0) and regulatory protein RhlR. The pubchem database (http://pubchem.ncbi.nlm.nih.gov) was used to download the 3D structures of both phytocompounds and natural auto inducers. All the molecular docking simulation analysis was performed using AutoDock platform.

## Results:

## Antibiogram profile of *P. aeruginosa*:

Out of 122 isolates of Pseudomonas strains, 80.3% (n=98) strains wereMDR & 19.6% (n = 24) of them were non MDR, susceptible to many antibiotics. Among the MDR strains 81.9% (n=100) of the strains were resistant to gentamycin, tobramycin, ciprofloxacin, levoflocacin, followed by amikacin 89.3% (n=109), ceftazidime 80.3% (n=98), piperacillin 79.5% (n=97), cefepime 74.5% (n= 91), while the lowest resistance was noticed in Imepenem 7.3% (n=9), dorepenem 9% (n=11) and Meropenem 11.4% (n=14).

## Antimicrobial effect of *B. diffusa* against *P. aeruginosa*:

**Figure 1:**
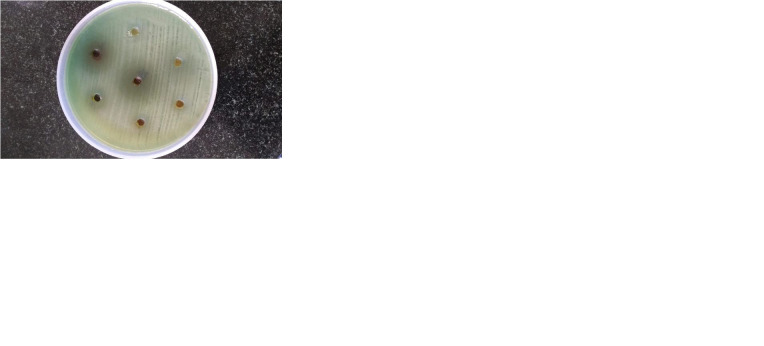
Agar well diffusion of plant extracts, Boerhaviadiffusa, 2. *Terminalia chebula*, 3. *Asparagus racemosus*, 4. Syzgium cumii, 5. *Azadiracta indica*, 6 & 7. Negative controls

Figure 1 describes the efficiency of plant extracts in treating Pseudomonas strains. Boerhavia diffusa, and *Syzgium cumini* plant extracts showed higher zone formation exhibiting a promising activity against the MDR strains of P.aeruginosa(Figure 1).The MIC analysis was performed for all the five plant extracts in a range from 0.0001% to 0.5% and corresponding values were monitored to calculate the percentage inhibition. While comparing the percentage inhibition values (Table 1), it was found that *Boerhavia diffusa* extract showed more efficacies against *P. aeruginosa*. Hence this plant extract was further taken for detailed experimental process including GC-MS, NMR, HPLC analysis and molecular docking analysis.

## Characterization of bioactive compounds:

**Figure 2:**
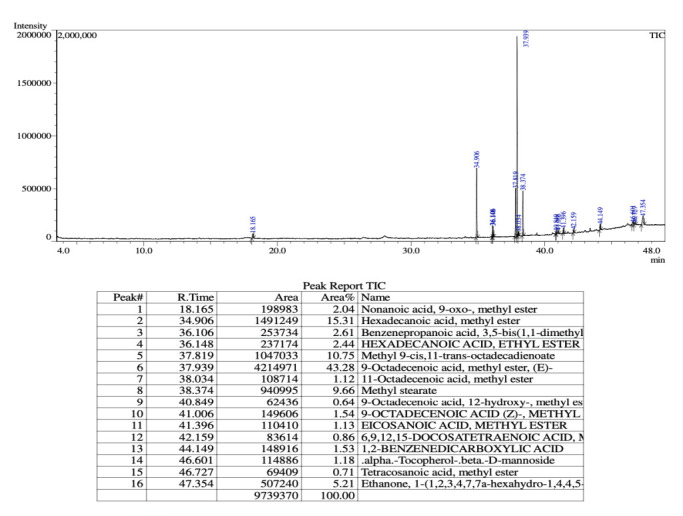
GC MS spectrum peaks and identified phyto compounds

**Figure 3:**
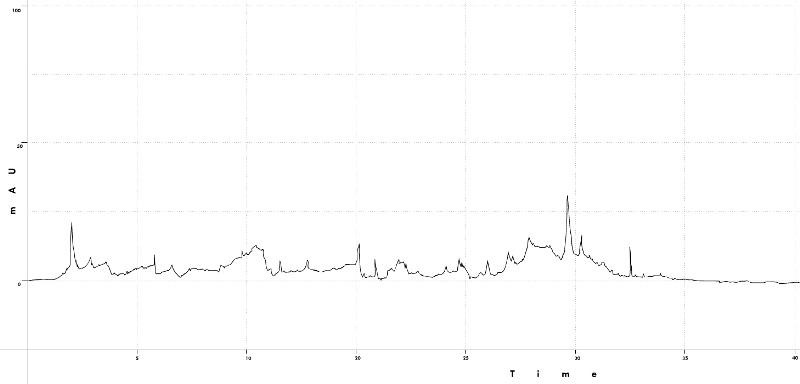
HPLC analysis for the *Boerhavia diffusa* Soxhlet extractions

**Figure 4:**
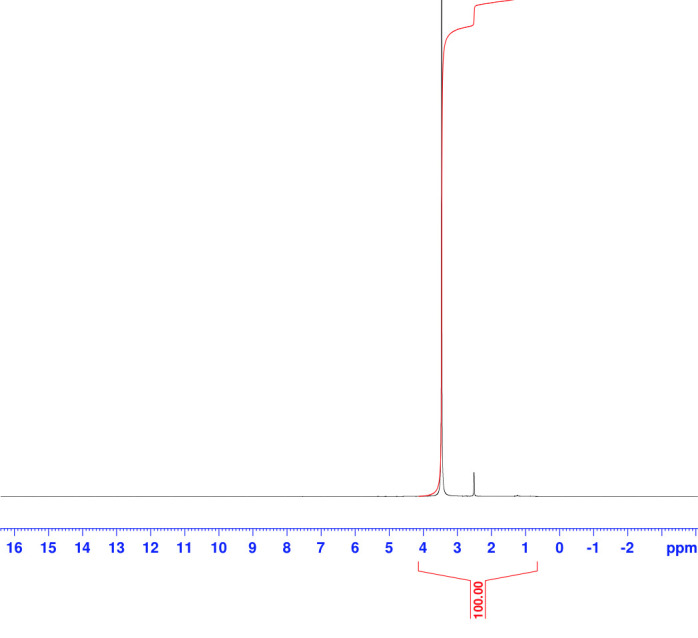
NMR analysis for the *Boerhavia diffusa* Soxhlet extractions

The GC-MS analysis revealed the presence of 16 various phyto-compounds in the methanolicextract of *Boerhavia diffusa* plant. Figure 2 describes all the 16 compounds details including their retention time, peak area, area with their names. Many notable peaks were observed in the retention time from 34 - 46 (Figure 2). diffusaSoxhlet extractions were analysed using HPLC at 260nm wavelength, maintaining a stable column temperature of 27°C. There were peaks observed at retention times 2.4334, 20.8243, 29.2714, 32.8861. A perfect resolution peak is observed at 29.2714±plusmn;0.005 (Figure 3).*Boerhavia diffusa Soxhlet* extractions were analysed using NMR analysis to find the specific compound, Boeravinone O (Figure 4).

## Molecular docking analysis of LasR Ligand binding domain bound to its auto inducer LasR (PDB ID: 2UV0) with the modeled compound:

**Figure 5:**
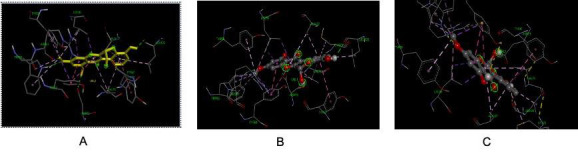
Molecular docking results; A - 3D view of protein-ligand interactions with amino acids residues; B - 3D view of protein-ligand interactions with ball and stick models; C - 3D view of protein-ligand interactions with Hydrogen bonds.

**Figure 6:**
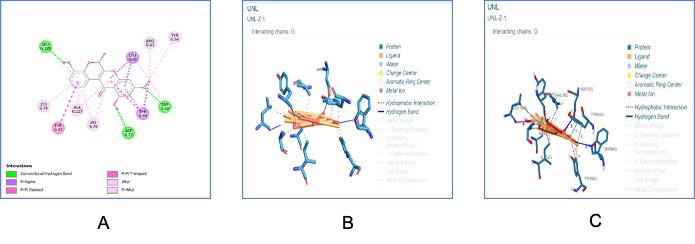
Ligand Interaction analysis; A - 2D interactions of ligand and protein molecules with amino acids residues; B - Interaction of ligand with chains G of protein; C - 3D view of interactions of all the points of ligands with protein chains G

**Figure 7:**
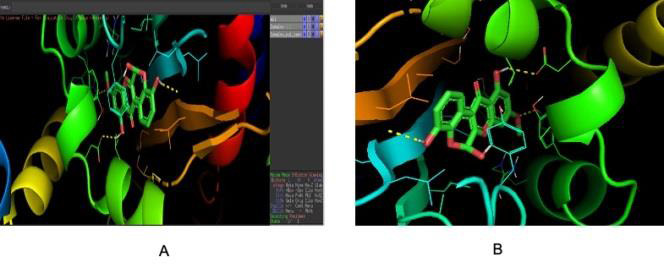
Pymol Complex view output file result; Figure 7 B: Close Pymol Complex view: Docked Binding complex of protein and Ligand. It describes the Pymol complex view with output file result and close Pymol complex view.

Docking analysis of the receptor Las R (LasR Ligand-binding bound to its auto-inducer of *P. aeruginosa* - PDB ID: 2UV0) with the modeled compound shows binding affinity with a least binding energy of -10.8kcal/mol. Quorum sensing of *P. aeruginosa* has been extensively studied and LasR is one of the key transcriptional regulators responsible for the production of toxic virulence factors. The bound ligand-receptor complex was further analysed for the amino acid interactions. Figure 5 and Figure 6 showed the 3D view protein -ligand interactions with amino acid residues and with ball and stick models (Figure 5,Figure 6,Figure 7). Each monomer of the dimerized, symmetric LasR-ligand binding domain contains a single, deeply buried ligand. The monomer fold is a five-stranded anti-parallel sheet sandwiched between three helices on either side. The 3oxoC12HSL autoinducer is buried from the solvent in a pocket formed between the sheet and helices 3, 4, and 5 and lies parallel to the sheet. Helix 6 forms the majority of the intermolecular H bonds and hydrophobic contacts on the opposite side of the -sheet, helping to create a significant dimer interface that engulfs 1900 square metres of surface area. LasR has a binding pocket of approximately ∼670 Å3 size. Figure 7 showed the 3D view of the protein ligand interaction with hydrogen bonds and showed the 2D interactions of ligand and protein molecules with amino acids residues. LasR has a large hydrophobic. Pocket formed by residues of Leu-36, Gly-38, Leu-39,Leu-40, Tyr-47, Glu-48, Ala-50, Ile-52,Tyr-56, Trp-60, Arg-61, Tyr-64, Asp-65, Gly-68, Tyr69, Ala-70, Asp-73, Pro-74, Thr-75, Val-76,Cys-79, Thr-80, Trp-88, Tyr-93, Phe-101,Phe-102, Ala-105, Leu-110, Thr-115, Leu-125, Gly 126, Ala-127 , and Ser-129. and depicts the interaction of ligand with chains G of protein. The LasR complexed with autoinducer (PDB ID: 2UV0) is housed within this hydrophobic pocket and it has established six intermolecular hydrogen bonding with the following aminoacids namely Tyr-56, Trp-60, Arg-61, Asp-73, Thr-75 and Ser-129. While the binding studies with our modelled compound indicate hydrogen bonding with Asp-73, Trp-60 and Leu-125 which indicates that our compound is also housed within the large hydrophobic binding pocket of LasR. The Table 2(see PDF) reveals the Summary of the molecular interaction of the ligand with 2UV0 and describes about the binding affinity and other details about hydrogen bond and hydrophobic interactions along with cation, anion and alkyl interactions. Table 3,Table 4(see PDF) depicts the details about the hydrophobic and Hydrogen bonds interactions of small molecules with proteins. Additionally, a π-π interaction with Tyr-47 and a π-σ interaction with Leu-36 and Tyr-64 were observed. Hydrophobic interactions were observed with the following amino acids residues Ala-50, Ile-52, Tyr-56, Tyr-64, Val-76,Leu-125 and Ala-127. Interactions with the alkyl group were observed with the amino acids Tyr-56, Arg-61, Val-76, Cys-79, and Ala-127. The different kinds of interactions observed between the modelled compound and LasR reveal that our compound effectively interacts with the binding of the transcription regulator LasR.

## Discussion:

Quorum sensing is very well known mechanism studied in *P. aeruginosa* behind its multi-drug resistance and biofilm formation. Additionally, studies have documented the secretion of important virulence factors including pyocyanin, rhamnolipids, exotoxins, protease and elastase aiding the formation and progression of biofilms. It is clearly observed in recent study that *P. aeruginosa* secretes N-Acylated L-Homoserine lactone (AHL) as the main component involving the initiation of LasR transcriptional regulator. Initiations of this regulator further lead to enhanced protein synthesize and lead to biofilm formation. It is well known that LasR has two major domain and among them one is natural inducer and the other one was OdDHL (N-3-oxododecanoyl-L-homoserine Lactone), and this is responsible for the conversion of monomeric protein into dimeric form. Various studies reported that targeting the QS pathway with plant based bio-active compounds is the key mechanism to target and arrest the biofilm formation. In a current report, they utilized the phytochemical contents from Avacado against *P. aeruginosa* PAO1. After performing various analytical experiments, they performed molecular docking for the LasR protein inhibiting the QS activity [9]. In another study, molecular docking reveals the action of mechanism and proved that bioactive compounds from Niaouli essential oil as a promising compound in inhibiting the LasR system [8]. In correlation with these, the compound Boeravinone Oin the present investigation holds good in targeting the LasR and further QS inhibition. Many reports suggested that *P. aeruginosa* possess the extensive mechanism of quorum sensing signalling molecules (QSSMS) or auto-inducer called acyl homoserine lactones (AHLs). It is very well established that these AHLs regulate the production of virulence factors along with antibiotic resistance and biofilm formation. Among the auto-inducers, LasI and RhI are well established in *P. aeruginosa*. Reports proved that adapting the silver nanoparticles to arrest the binding of LasR and Rh1R to its receptor site and inhibition of biosynthesis of S-adenosyl methionine (SAM) (Syed Ghazanfarali et al. (2017) that had led to the reduction of AHL synthesis and inhibition of las l/Rh1l synthase. In-silico based docking studies also had documented AgNPs bound to the active site of Las l/Rh1l and lasR/Rh1R of the proteins had the property to inhibit the quorum sensing in *P. aeruginosa* [10]. With this in background, the present investigation had analyzed the *Boerhavia diffusa* (*B. diffusa*)for its QS inhibiting property. Various reports demonstrated the importance of the different phytochemical compounds from diverse natural sources to act against the *P. aeruginosa* QS system. All these reports proved the efficiency of the different plant compounds in inhibiting the mechanism of biofilm formation in *P. aeruginosa* by targeting specifically QS system using analytical experiments and molecular docking studies [12, 13,14,15, 16,17]. Hisham Abdel Monemabbas et al. (2018). Umamaheshwari et al. studied qualitative analysis of phytochemicals and their antimicrobial activity using extracts of *B. diffusa* in different solvents such as methanol ethanol, showed wide range of antimicrobial activity against major pathogens like S. aureus and P. [13]. Similar studies conducted by Ramachandra et al, using extracts prepared from *B. diffusa* roots and aerial parts by agar well plate methods expressed strong antibacterial property [14]. Study conducted by Kaviya M et al, 2022,found the highest zone of inhibition of about 8mm in diameter using decoctions of *B. diffusa* leaves and stems, as well as root ethanol extract, at 200 µg concentration, against the *P. aeruginosa*. GCMS analysis of their study with root ethanolic extract revealed alkaloids, phenols, flavonoids which have vast medical and therapeutic applications. (Kaviya, M.2022)*B. diffusa* has been examined extensively for presence of pharmacological qualities and many bioactive substances [15]. Molecular docking studies conducted by Kaviya M et al, using *Pseudomonas aeruginosa* quorum sensing protein PqsR, demonstrated top ranked molecules featuring Tyr258, Arg209, Ile236 and Leu197. Their results revealed good binding affinity with crude extract exploring antibacterial activity against *P. aeruginosa* (Kaviya M et al, 2022). There was not much report to understand the mechanism of inhibiting the QS activity of *P. aeruginosa* PAO1by utilizing the B.diffusa extract. After analysing a range of plant extracts, we observed that Boeravinone of *B. diffusa* plant extract showed significant results in antibacterial susceptibility test followed by which molecular docking analysis was performed and confirmed that the binding of ligand protein interaction is promising enough to inhibit the formation of QS activity.

## Conclusion:

As many studies suggest, the inhibition of the Quorum sensing (QS) is known to be the harmless process and highly productive process in arresting the infinite number of harmful pathogenic bacteria. Our report clearly depicts the high potential of *B. diffusa* and its various bioactive compounds in arresting the QS dependent virulence pathways and components and the process of biofilm formation in *P. aeruginosa*. Molecular docking results also states that protein-ligand interaction reveals the importance of this plant extract in treating *P. aeruginosa*. Thus, all these results and data clearly provide information unraveling their disease pathogenesis mechanism and establishment of productive anti-infective agents against *P. aeruginosa* infections.

## Figures and Tables

**Table 1:** Comparative Analysis of MIC for five different plant extracts

**PE Name**					**PEC**					
		**0.0001% PE**	**0.0005% PE**	**0.001% PE**	**0.005% PE**	**0.01% PE**	**0.05% PE**	**0.1% PE**	**0.5% PE**	**Control**
*Boerhavia diffusa*	PA Strain (OD±SD)	0.617±0.01	0.421±0.01	0.232±0.009	0.080±0.006	0.073±0.01	0.729±0.005	0.065±0.008	0.056±0.01	0.812±0.007
	Percentage Inhibition	23.975	48.049	71.312	90.028	91.004	91.011	91.945	93.071	
Azadirachtaindica	PA Strain (OD±SD)	0.623±0.007	0.577±0.001	0.533±0.012	0.404±0.016	0.217±0.015	0.177±0.006	0.154±0.001	0.153±0.02	0.702±0.019
	11.232	17.762	21.155	42.412	69.033	74.716	78.049	78.065	11.232	
*Asparagus racemosus*	PA Strain (OD±SD)	0.641±0.013	0.551±0.09	0.15±0.12	0.143±0.16	0.139±0.09	0.125±0.1	0.122±0.11	0.120±0.08	0.716±0.011
	Percentage Inhibition	10.466	23.027	79.014	80.026	80.582	82.439	82.919	83.211	
Syzygiumcumini	PA Strain (OD±SD)	0.560±0.01	0.470±0.009	0.219±0.016	0.089±0.012	0.066±0.011	0.062±0.015	0.053±0.01	0.053±0.017	0.693±0.014
	Percentage Inhibition	19.162	32.097	68.273	87.037	90.375	90.911	92.304	92.317	
Terminaliachebula	PA Strain (OD±SD)	0.6±0.0012	0.545±0.017	0.441±0.01	0.364±0.016	0.232±0.009	0.154±0.015	0.135±0.015	0.135±0.017	0.675±0.012
	Percentage Inhibition	10.974	19.117	34.641	46.054	65.551	77.071	79.909	79.915	
